# Dynamic quarantine: a comparative analysis of the Chilean public health response to COVID-19

**DOI:** 10.1017/S0950268820002678

**Published:** 2020-11-04

**Authors:** Gonzalo Grebe, Javier A. Vélez, Anton Tiutiunnyk, Diego Aragón-Caqueo, Javier Fernández-Salinas, Mónica Navarrete, David Laroze

**Affiliations:** 1 Instituto de Alta Investigación, CEDENNA, Universidad de Tarapacá, Casilla 7D, Arica, Chile; 2 Escuela de Medicina, Universidad de Valparaíso, Valparaíso, Chile; 3 Escuela de Administración y Negocios, Facultad de Administración y Economía, Universidad de Tarapacá, Casilla 7D, Arica, Chile

**Keywords:** Chile, COVID-19, daily transmission rate, public health response, quarantine

## Abstract

In this study, an analysis of the Chilean public health response to mitigate the spread of COVID-19 is presented. The analysis is based on the daily transmission rate (DTR). The Chilean response has been based on dynamic quarantines, which are established, lifted or prolonged based on the percentage of infected individuals in the fundamental administrative sections, called communes. This analysis is performed at a national level, at the level of the Metropolitan Region (MR) and at the commune level in the MR according to whether the commune did or did not enter quarantine between late March and mid-May of 2020. The analysis shows a certain degree of efficacy in controlling the pandemic using the dynamic quarantine strategy. However, it also shows that apparent control has only been partially achieved to date. With this policy, the control of the DTR partially falls to 4%, where it settles, and the MR is the primary vector of infection at the country level. For this reason, we can conclude that the MR has not managed to control the disease, with variable results within its own territory.

## Introduction

The COVID-19 pandemic that began in Wuhan, China, in December 2019 [1] has rapidly spread to the rest of the globe during 2020, reaching unprecedented proportions. As of 15 May 2020, 4 338 658 cases have been confirmed, with 297 119 deaths [2]. Contending with this novel threat is a challenge that every country with confirmed cases must face. Given the broad spectrum of diverse public health strategies and initiatives taken by each country, it is worth addressing the effectiveness and efficiency of such measures.

Some countries have managed to control the pandemic through specific strategies [3] that have significantly decreased the daily transmission rate (DTR), defined as the daily percentage of growth of the cumulative confirmed cases, to values close to 0.2%. Of course, a nominal zero is very hard to achieve, since there is still no vaccine or treatment that would allow the complete eradication of the disease. However, it is worth mentioning that great efforts are being made by the scientific community to find an effective treatment for COVID-19 [4, 5]. In this context, three countries have reached a 0.2% DTR, South Korea, Germany and New Zealand [6], as of the 15 May when this analysis was being performed. All of them have very different but equally effective policies, and they will be taken as a reference for our analysis.

The implementation of control measures disrupts the normal functioning of a country in terms of the health, economic, social and psychological conditions of their population [7–11]. At this time, controlling the spread of the virus is a state priority in every country with confirmed cases. Indeed, low- and middle-income countries cannot adopt some of the measures that high-income countries have because resource availability and country preparedness vary dramatically [12]. Therefore, in this paper, the response of a developing country, Chile, is analysed. This country has adopted a mixed control strategy, using relatively massive testing and dynamic quarantines [13, 14]. Herein, dynamic quarantine is defined as the Chilean strategy of locking down specific neighbourhoods of certain cities based on the number of active cases in the territory. This lockdown is then weekly reassessed and lifted, prolonged or expanded as a function of these active cases. However, specific cutoff points for such measures are not clearly established, and decision-taking relies heavily on the Ministry of Health.

With this being said, this paper focuses on the Chilean public health response, to detect the advantages and shortcomings of the strategy in improving disease control and to compare Chile's experience to that of countries that have already relatively controlled the progression of the disease. This analysis could guide other countries in implementing a particular dynamic quarantine strategy, leaving the groundwork for the various stages of the pandemic's spread at the country level and then set parameters for international success. The objective is to calculate the degree of efficiency of the dynamic quarantine strategy applied in Chile. The primary indicator to determine this is the DTR, measured as a percentage, together with the cumulative number of confirmed cases, among other indicators.

The paper is organised as follows: ‘Methods’ section provides a description of the viral spreading stages. ‘Dynamic quarantine strategy’ section analyses the case of Chile in detail. Finally, the conclusions drawn from the results are presented in the section ‘Final remarks’.

## Methods

**Fig. 1.**
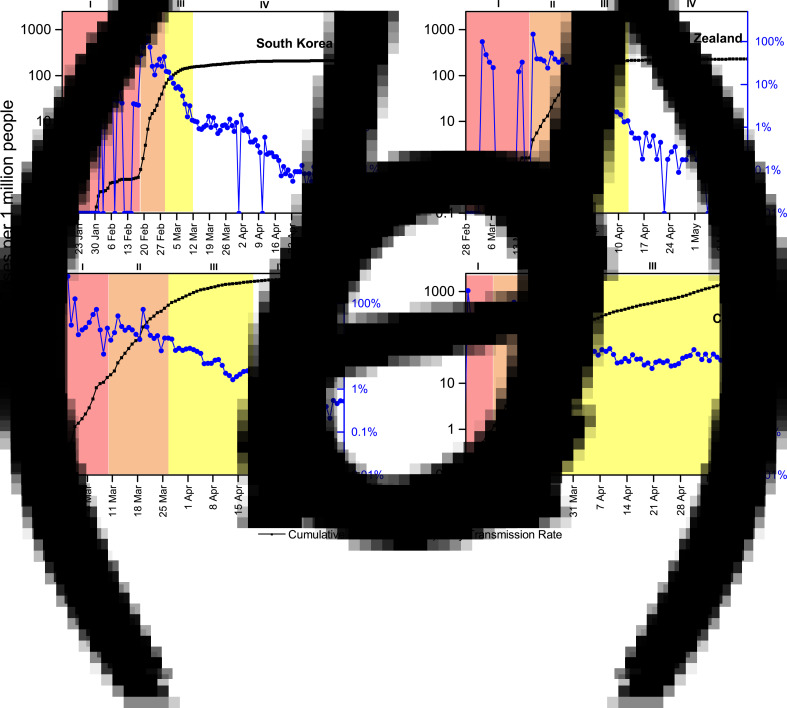
Temporal evolution of the total number of cases per million people (in black squares, logarithmic scale) and the DTR (in blue circles) from the first reported case in each country until 15 May. The different stages of the pandemic progression are colour coded as described above. (a) South Korea, first reported case on 20 January, (b) New Zealand, first reported case on 28 February, (c) Germany, first reported case on 26 February And (d) Chile, first reported case on 3 March.

Through the analysis of the available country-level data, four stages in the evolution of the pandemic can be identified. These stages are established to facilitate the understanding of the behaviour of the outbreak based on the DTR. For this measure, an empirical approach was taken, and countries with successful experiences fighting the pandemic, such as South Korea, Germany and New Zealand, were chosen as the leading example. From these, some patterns on the behaviour of the outbreak that consistently repeats across countries were identified, as shown in Figure 1. Based on these observations, we propose the following stages: the stochastic uptake stage, exponential growth phase, intervention phase and finally a new growth regime. (I) The first stage has been defined as the *stochastic uptake*, in which few cases appear and can be seen like an incipient stage. This is the period at which the virus is just arriving in a particular country and where cases are emerging slowly, sometimes parcelled out over time. This process can be assumed to be a problem of misinformation and a lack of established control, where there is likely to be an underestimation of the real cases. (II) The second stage is the *exponential growth phase*. Here, the disease settles in the country, cases start to rise exponentially fast and at the same time, initial control measures begin to be established as part of the state policy. However, there is not yet an established or ongoing response strategy, and a rapid advance of the virus is detected, with DTRs above 20%. (III) Then comes the strategic phase, which is called *intervention*. In this stage, the states implement their control strategies and begin to flatten the infection curve to some extent. Normally, if the strategies are correctly implemented, after a relatively short to medium length of time, a drastic decrease in the rate of infection should be observed. The beginning of this stage is marked by the last significant peak in the DTR before it starts a consistent and gradual decrease. (IV) When the strategies have been well established, a *new growth regime* appears in the confirmed cases, in which a relative control can be reached. At this point, the DTR drops below 2% and can be as low as 0.1%. This threshold was set empirically. In the leading examples mentioned above, once the country reached this 2% DTR, it did not surpass it again during the time considered in this analysis. In stage IV, the disease has been successfully contained. However, in the absence of a vaccine or effective treatment, it does not disappear, and there will always be a remnant. Therefore, it should be expected that COVID-19 will be a new constant pathology that will be present in the healthcare setting in the medium to long term [5]. Of course, it should be noted that if countries' strategies change, possible re-emerging outbreaks and increased rates of contagion may occur. A more detailed analysis of the countries is available in the Supplementary material. It is important to remark that Chile has not achieved the relative control stage because, as can be observed in Figure 1, the strategies implemented have not been able to decrease the DTR below the limit of 2% DTR, and it has remained above this mark.

## Dynamic quarantine strategy

In Latin America, there was more time and information available to set up a response. The first case was reported in Brazil on 26 February 2020 [15], 57 days after the WHO was first notified [16]. However, Brazil has a lower hospital capacity and nationwide preparedness compared to developed countries [17], making timely containment of the pandemic even more necessary. With the information available at the time, different strategies were implemented. In the cases of Argentina, Bolivia, Colombia and Peru, total quarantine was established when the total number of confirmed cases was still low. Some of these countries have been more successful compared to others in containing the DTR using this classic method. However, this will be left out of the analysis of this paper.

A unique case among the responses in Latin American countries was Chile. This country took partially timely actions, with a mixed strategy. Chile implemented mass testing, which resulted in a continuous increase in its testing capacity. This grew from 0.2 tests per 1000 individuals on the 8th of April to 0.6 on the 15th of May, making it the country in Latin America with the highest testing capacity per individual [18]. However, it is crucial to highlight that this testing capacity was not as massive as the testing capacity in higher-income countries. Public, private and university laboratories were in charge of the testing effort. The distribution of testing was concentrated in the most populated urban areas, and the Metropolitan Region (MR) was the region with the most tests performed [19]. In fact, the MR holds 65% of the total tests performed at the national level between 9 April and 15 May.

Unfortunately, it is not possible to accurately track the tests performed by region, since some samples were processed at a different region from where the sample was originally from. From this, if a case is confirmed, then the case is counted as confirmed case in the region from which the sample was originally from; however, if the test is negative, then it is counted as a test performed at the region where the sample was processed. Let us also remark that the average daily testing was 39 tests per 100 000 persons in the aforementioned time window [19]. In the present study period, a confirmed case is defined as a person with symptoms and a positive reverse transcription polymerase chain reaction result, and testing policy allows only individuals with symptoms to access such tests [20]. On the other hand, part of the strategy was to close educational establishments early, to close all nonessential business and to quickly declare a state of emergency, with curfew declared between 10 pm and 5 am [21, 22]. This partially restricted the mobility of people for several focused hours at night and prevented large gatherings during that period. In addition, in the capital, Santiago, the government implemented a strategy of quarantine divided into sectors delimited by administrative boundaries or communes, which are the fundamental administrative units, and even further dividing those communes into sectors and isolating a specific geographic location. However, entire cities or territorial regions were not quarantined, with the exception of Easter Island. The MR corresponds to one of the 16 regions that divide Chile. It is located at the centre of the country and includes the city of Santiago, the capital city of Chile and the surrounding areas, limited by the Andes Mountains. The dynamic quarantine was mostly implemented in MR which holds 8 125 072 people, accounting for approximately 42% of the country's population [23]. This makes the MR the main focus of confirmed cases in the country, as of 15 May, accounting for 74% of the confirmed cases [23]. Its high population density of 527.5 people per km^2^ [23] makes for a great challenge in containing the spread and might be a risk factor for a faster progression than other regions of the country with a lower population density have had. Panel d of Figure 1 shows the progression of the outbreak in Chile. It shows a very short *stochastic uptake stage* from the first reported case on 3 March to 17 confirmed cases on 10 March. From 11 March to 27 March, *an exponential growth stage* is established with 1610 cases in 16 days. The stage where the effect of the control strategies started to show begins on 27 March, and more than a month later, Chile had only reached an 8% DTR, without being able to reach a stage of the *new growth regime* with relative control (under the 2% mark that the *successful* countries show). In fact, the DTR shows many fluctuations.

Let us note that the number of cases was not significantly high at the time of implementation of control measures. The dynamic quarantine effectively began on 27 March, when the number of cases was already at the peak of 1610. However, when compared to the case of New Zealand, this can be considered late, as the measures in that country were applied at 283 cases. The Chilean strategy began only 1 day after New Zealand's (26 March), but while in New Zealand the DTR reached 1% in 15 days, Chile's only reached 5.2% in the same 15 days. The lowest ever reached has been 3.1%, and from there, it has increased to 8%. In fact, the DTR has the possibility of further increasing.

### Analysis of the intervention phase in Chile

**Fig. 2.**
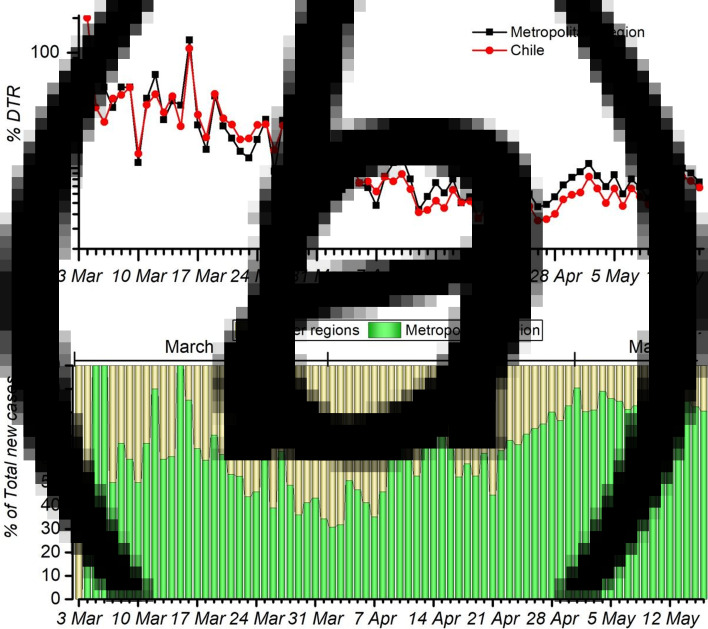
(a) Temporal evolution of the DTR from the first reported case on 3 March until 15 May of 2020 in Chile and the MR. (b) Percentage of new confirmed cases, colour-coded green for the MR and yellow for the other regions.

When analysing the state of the pandemic in Chile, it can be clearly seen that the *effect of the strategies* has shown a prolonged intervention phase, without giving way to a *new growth regime* with relative control, which is defined as a DTR under 2%. Panel a in Figure 2 shows the temporal evolution of the DTR between 3 March and 15 May 2020 for the country and for the MR, the region that accounts for the great majority of confirmed cases. It can be seen that the country's trend is the same as for the capital, since they are highly correlated. In fact, Pearson's correlation coefficient of both time series, discarding the data before the strategy, is 0.978. It is noted that on average, the MR has 74.8% of the country's confirmed cases. From panel b of Figure 2, it can be seen that the MR owns considerable blame for the spread in the last month, representing 82% of the new confirmed cases at the national level as of 15 May. Clearly, it can be inferred that the MR is the main focus of infection in the country.

Table 1 presents the Chilean statistics separated by region of the country, ordered from north to south. It is evident that in the northern and southern regions, the virus has spread differently than in the central regions of the country. It is important to highlight that the southernmost region, *Magallanes*, has the highest percentages of confirmed cases per population and deaths per population. This ratio clearly differs from the numbers of the other regions of Chile. Further developing on this, the southern areas besides *Magallanes* have had a much lower percentage of cases per population than the Central and Northern regions. The Northern regions present somewhat similar situation of cases per population, except for *Atacama* and *Coquimbo*, which show substantially lower percentages. On the other hand, as noted above, the Central region has the majority of the country's cases. However, the Central region does not differ as dramatically from the rest of the country in deaths per population as it does in the number of confirmed cases. Chile has a unique geography, being more than 4200 km long and separated by the Andes Mountains from its neighbouring countries. Land connectivity is relatively poor, with long distances between cities. Therefore, the country is relatively isolated by land: from other countries by the Andes mountain range and within the country by large separations between regions. This might account for the dramatic differences in confirmed cases between the different regions of the country. Let us remark that there is a highly unequal distribution of hospital capacity, critical beds and physicians across the different regions of the country, being the central regions, specifically the MR, that holds the majority of the health resources of the country [24–26]. In fact, physicians' availability per 100 000 in the MR is almost double the availability of physicians per 100 000 in the northern and southern regions [27]. This might account for the considerable differences observed between the MR and other regions of the country regarding deaths relative to the number of confirmed cases. However, further studies on the subject are required to draw such conclusions. The maps of Chile seen in Table 1 represent in a colour code the number of accumulated cases as well as the number of deaths. From them, we can clearly observe the distribution of the pandemic in Chile. Since MR holds the most critical responsibility for the country's propagation of the virus, let us study its strategy.

**Table 1.** Discrete data by region and its proportional rate per inhabitant as of 15 May [23]

Region	Population	Total cases	Cases/population (%)	Total death	Death/population (%)	Cases	Deaths
Arica y Parinacota	252 110	368	0.146	7	0.003	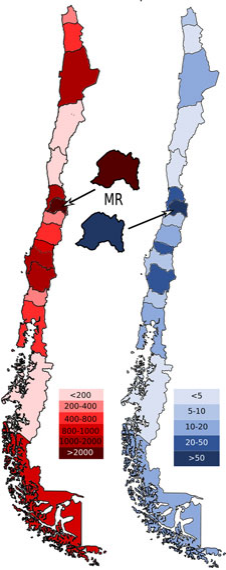	
Tarapacá	382 773	779	0.204	2	0.001		
Antofagasta	691 854	1331	0.192	12	0.002		
Atacama	314 709	143	0.045	0	0.000		
Coquimbo	836 096	180	0.022	1	0.000		
Valparaíso	1 960 170	1312	0.067	27	0.001		
MR	8 125 072	29 276	0.360	221	0.003		
O'Higgins	991 063	323	0.033	5	0.001		
Maule	1 131 939	540	0.048	14	0.001		
Ñuble	511 551	885	0.173	20	0.004		
Biobío	1 663 696	1036	0.062	9	0.001		
Araucanía	1 014 343	1564	0.154	43	0.004		
Los Ríos	405 835	219	0.054	6	0.001		
Los Lagos	891 440	641	0.072	11	0.001		
Aysén	107 297	8	0.007	0	0.000		
Magallanes	178 362	937	0.525	16	0.009		
Total	19 458 310	39 542	2.16	394	0.031		

### Strategy in the Metropolitan Region

Health authorities, along with the Ministry of Health (MINSAL), quickly discarded the idea of total quarantine and opted to apply a strategy of partial quarantines in the capital starting 27 March [21]. Dynamic quarantine follows weekly reevaluation and patterns, where the quarantine is established, prolonged, or lifted, depending on the relative number of cases in each *commune*. The MR has 52 administrative subdivisions called communes, 18 of which are rural. The vast majority of the communes are concentrated in the capital city, usually called *Great Santiago*. It hosts 32 of the densely populated communes, including the *commune of Santiago*, which is essentially the downtown, usually called *Santiago Centro*. In the following, when we refer to Santiago, it will be the Great Santiago, whereas when we refer to Santiago Centro will be the commune of Santiago. Since Santiago is a large city, its quarantine plan was divided geographically according to these communes and by the proportion of infected inhabitants. It is important to note that the divisions used in the government's strategy were based mainly on fundamental administrative divisions, not on territorial or macro-level divisions. Only on a long holiday or during the weekends, where the high flow of vehicles was anticipated, were sanitation cordons established between one region and another, in order to prevent a massive outflow of people from the capital to the surrounding central regions.

**Fig. 3.**
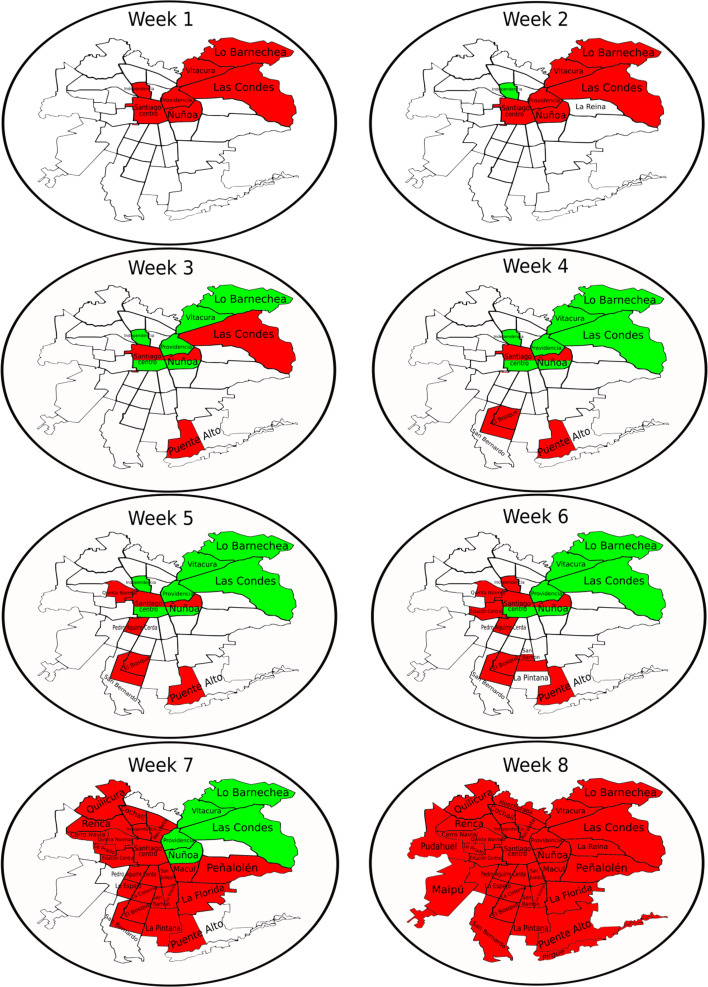
Spatiotemporal evolution of the quarantine in MR within the week as a time scale and starting on 31 March. Red (dark) colour represents communes with quarantine. Green (light) colour represents communes that whose quarantine has been lifted. White colour represents communes without quarantine.

Figure 3 shows the spatiotemporal evolution of the communes through the dynamic quarantine approach. We can observe that in the beginning, some of the communes entered quarantine in the central and northwest areas of MR, from the geographical point of view, which include Santiago Centro. The northwest part is the wealthy area in Chile, which was the first area with positive cases. One week later, only one commune was left of the quarantine. Then, some of them entered quarantine, and others went out for 5 weeks. Finally, the whole MR was put under quarantine in the 8th week. More precisely, the first communes that entered quarantine did it as a function of the proportion of confirmed cases per population, with *Vitacura*, *Las Condes*, *Ñuñoa*, *Lo Barnechea*, *Providencia*, *Santiago Centro* and *Independencia* being the first quarantined. Initially, before the government took any quarantine measures, these had a proportion of confirmed cases per population of 0.044% on average, while the communes that did not enter quarantine had a proportion of 0.008%. However, in the particular case of *Independencia*, this proportion was 0.011%, which is close to the average of the communes without quarantine. It is important to note that the data provided by MINSAL [23] with detail at the commune level are from 30 March. Epidemiological reports with detailed data are not published on a daily basis but rather every 2 to 5 days. For this reason, the following analysis uses the dates of the epidemiological reports available.

**Fig. 4.**
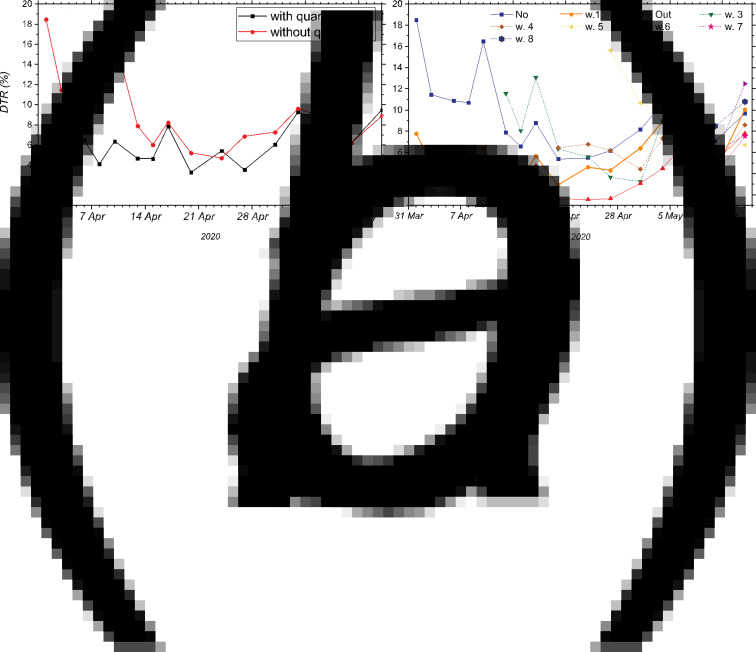
Percentage increase in the DTR as a function of time in the MR from 30 March until 15 May. (a) Communes with quarantine (squares) and without quarantine (circles). (b) Segregation into dynamic quarantines: communes that did not enter quarantine are represented by squares (No). Communes that entered quarantine during the first week of quarantine measures and remained under quarantine are represented by circles (w.1). Communes where quarantine was lifted are represented by the top-pointing triangles (Out). Communes that entered quarantine in weeks 3, 4, 5, 6, 7 and 8 are represented by different markers (abbreviated w.3, w.4, w.5, w.6, w.7 and w.8, respectively).

Let us analyse the temporal evolution of the DTR in the MR. In panel a of Figure 4, one observes that the set of communes without quarantine has a higher DTR, reaching levels of 18% at the peak. However, as days pass, it decreases until, during mid-April, it has relatively similar DTRs to those of communes under quarantine. In turn, in the communes that were under quarantine at that time, it can be observed that on 1 April, after 4 days of applying the restrictive measure (starting 27 March), the DTR drops to 8% and further decreases to its nadir of 3% on 8 April. However, it soon increases to 9% to 15 May. From 24 April to 15 May, it can be seen that the curves are correlated and that the trends are somewhat similar. Therefore, it is worth asking whether the dynamic quarantines had any effect at all compared to no quarantine. In view of this result, it is necessary to separate the quarantined communes from those that left the quarantine at some point and not to compare the latter with the communes that were never quarantined. As mentioned above, the strategy is called dynamic quarantine because MINSAL lifted the quarantine in communes where it observed a decrease in the number of cases. Therefore, it becomes necessary to isolate these communes in the comparative analysis, since adding them to the communes that were never quarantined alters their DTR value.

Now, let us separate the communes into those that were never quarantined, those under quarantine in the week at which the measure was taken and those where the quarantine was lifted at that time. The communes that enter quarantine as time progresses are represented by the week, they first entered quarantine. As can be observed in panel b of Figure 4, it is essential to highlight the trends observed in the communes that never entered quarantine (squares), the communes that entered quarantine in week 1 and whose quarantine was not lifted (circles) and finally, the communes that entered quarantine but later left it (top triangles). They are also marked as *No*, *Out* and w.1, respectively. The set of communes that entered quarantine in week 1 (w.1) had a relative DTR difference of 10% from the set without quarantine. The w.1 group reached a 3% daily infection rate on 13 April. Therefore, the effect of quarantine was positive. However, on that date, the authorities decided to lift the quarantine in some communes (Out). It can be observed that they reached the lowest DTR overall, achieving 2% at some point, although they did not manage to continue with this trend. The w.1 communes that stayed on quarantine on 17 April are further divided into geographical areas from North to South, in which quarantine is lifted in some and prolonged in others, thus making the administrative barrier even weaker. For this reason, a consistent increase in the DTR of these communes can be observed since the day they were further separated, arriving at DTRs fairly similar to those of communes that were never under quarantine on 15 May. It is important to note that although some geographical area of the commune was not under quarantine, the commune as a unit is still considered *under quarantine*. The communes without quarantine (‘No’ – squares) show an initial decrease in the DTR, from 18% to 11% on 8 April. At that moment, the authorities decided to establish quarantines in new communes. These communes are represented by w.3. This further decreases the DTR of communes without quarantine, reaching nearly a 5% DTR on 20 April. However, after this, it goes back up to 11% on 15 May. As time progresses, other communes enter and leave quarantine. However, in the overall picture, it can be observed that the implemented strategy was not useful in reaching a relative control with a *new growth regime*, emphasising that restrictive measures with permeable borders do not work. This may be due to multiple factors, such as movement for work, noncompliance with regulations, the lack of clear demarcation of administrative borders and many other possible factors, that prevented this dynamic strategy from achieving the desired relative control. For example, there was no strict restrictions on movement between communes, in the sense that with a permit issued by the police, it was possible to carry out shopping groceries, personal administrative procedures and even visit other communes (regardless if the commune was under quarantine or not). This permit could be obtained online up to five times a week, with a duration of 4 h. Besides, there were collective work permits. There was no restriction for workers from communes without quarantine to go to work to communes with quarantine and vice versa, every day, for any essential service such as supermarkets, gas stations, delivery and public transportation and hospitals.

**Fig. 5.**
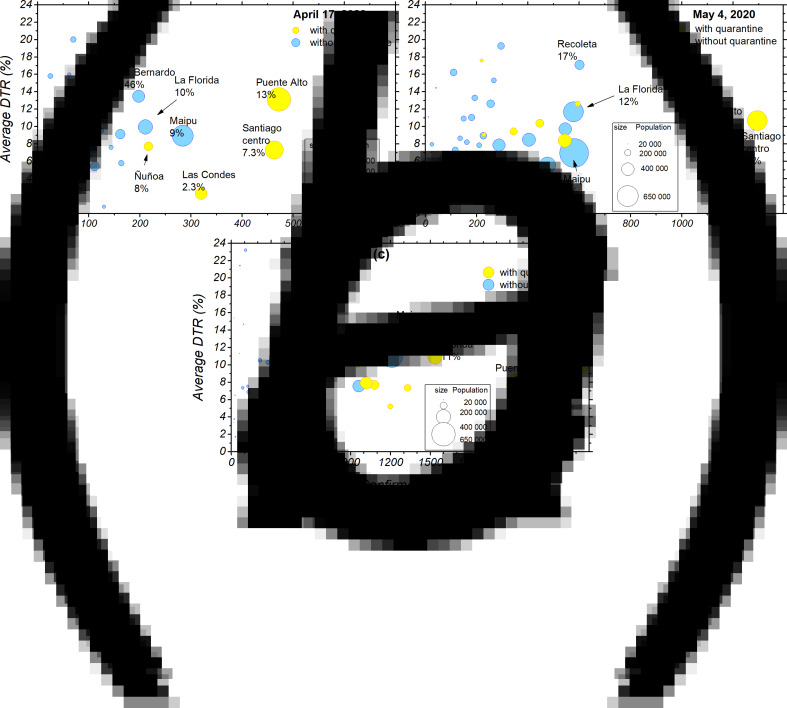
Progression of the confirmed cases, population and DTR for the different communes from 17 April to 15 May. (a) Average DTR as a function of the number of confirmed cases according to commune population size as of 17 April. Communes that were in quarantine are represented in yellow circles, and communes that were not under quarantine are represented by blue circles. (b) Average DTR as a function of the number of confirmed cases according to commune population size as of 4 May. (c) Average DTR as a function of the number of confirmed cases according to commune population size as of 15 May.

Figure 5 shows the average DTR as a function of the number of confirmed cases from 27 April to 15 May. The graph shows the details by commune. Each circle represents a commune in the MR, such that the area represents the number of inhabitants in that specific commune. The light-blue colour stands for the communes that were not quarantined on the date indicated, while the light-yellow colour represents the communes that were quarantined. It can be observed that communes such as *Maipu*, *La Florida* and *Puente Alto*, with large populations, initially show considerably high DTRs. In the particular case of *La Florida,* a commune with approximately 400 000 inhabitants, on 17 April (panel a), it had a DTR of 10%, and on 4 May (panel b), it had 578 confirmed cases and a DTR of 12%, but it was still not put under quarantine. On the other hand, *Ñuñoa*, a commune with approximately 250 000 inhabitants, on 4 May had 365 confirmed cases with a DTR of 3.6% and was under quarantine. It can be established that by 4 May, *La Florida* had approximately three times the DTR and 160% the population of *Ñuñoa*, but quarantine was never established. Similar cases can be observed in *Maipú*, where it can be observed from 17 April until 15 May that it consistently showed higher DTRs, had a greater population and a larger number of confirmed cases than *Ñuñoa* but nevertheless did not enter quarantine (panels a–c). Thus, if in the large communes continue to have quickly increasing numbers of confirmed cases, they will likely have a hospital saturation.

**Fig. 6.**
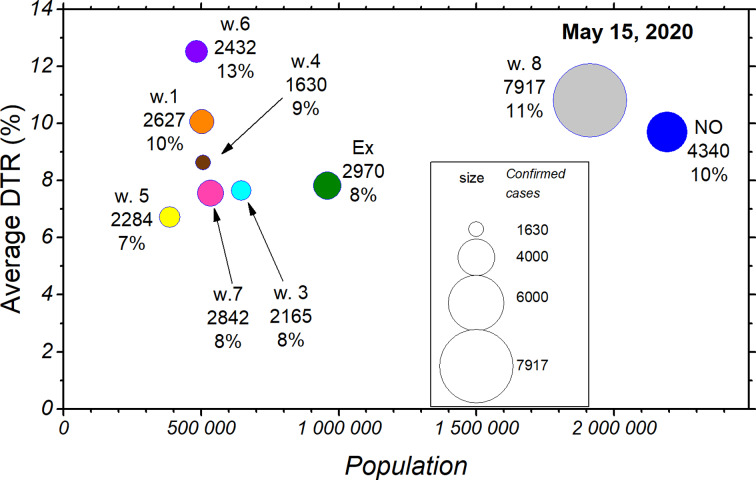
The situation by commune in terms of DTR (% daily increase) and number of people, separated by the week at which they entered quarantine or did not enter it at all. The area of a circle indicates the corresponding number of confirmed cases.

Finally, Figure 6 shows the average DTR on 15 May as a function of the population. The size of each circle indicates the corresponding number of confirmed cases. From the graph, it can be observed that the communes without quarantine (in blue) show high DTRs, averaging 10%, but with a much higher potential of influencing the overall picture, since they represent close to 2.2 million inhabitants. This makes them an essential focus of confirmed cases, and considering the rapid spread of the disease and how these communes are in close contact with a larger world, they could potentially influence the trends observed both regionally and nationally.

## Final remarks

From the overall results, it can be established that a dynamic quarantine strategy works partially, stabilising the DTRs between 4% and 11%. For this reason, it does not succeed in achieving relative control of the pandemic at a national level. It is also essential to note that communes that did not enter quarantine maintained high levels of DTRs, which fluctuated from 5% to 18%. On the other hand, a total quarantine of single communes was initially effective at the communal level, reaching levels of relative control in four of the seven communes under quarantine by that time. This was observed in the communes of *Vitacura*, *Las Condes*, *Lo Barnechea* and *Providencia*. However, as discussed above, when the quarantine in these communes was lifted, they quickly escalated to similar DTRs as their fellow communes that were never on quarantine. It is important to add that communes under partial quarantine experienced a decrease in the DTR. Nonetheless, they have not reached the level of relative control, showing DTRs that fluctuate between 4% and 10%, as seen in *Ñuñoa*, *Santiago Centro*, *Puente Alto*, *San Bernardo* and *El Bosque*. It is also important to note that communes that quickly are drawn out of quarantine without consolidated low DTRs can quickly revert to high DTRs, as was seen in *Independencia* and *Lo Barnechea*, both of which reached the 2% mark for only 1 day and then climbed back up to high DTRs; as of 15 May, their DTRs are 5%. The results show the insufficient effectiveness of the dynamic quarantine strategy at the aggregate regional level since, while the DTRs of some communes decreased, others continued to rise. Consequently, it is prudent to conclude that the DTR stagnates due to the combined result of the previously exposed points: there are many communes without quarantine, communes with partial quarantine and communes that leave quarantine too early and relapse soon after. It is also crucial to the point that only three communes (*Vitacura*, *Las Condes* and *Providencia*) maintained consolidated low DTRs. Nevertheless, they also relapsed in the long term. Finally, when the decrease in the DTR stagnated in the MR, it also was correlated with the situation at the country level to stagnate. Therefore, the dynamic quarantines in the MR could explain why it has not been possible to control the pandemic in Chile so far, exposing the inefficacy of the dynamic quarantine strategy.

## Data Availability

The data that support the findings of this study are available from the corresponding author upon reasonable request.

## Supplementary material

For supplementary material accompanying this paper visit https://doi.org/10.1017/S0950268820002678. click here to view supplementary material
