# Supplementary material for: Dynamic quarantine: a comparative analysis of the Chilean public health response to COVID-19
Source: Epidemiol Infect. 2020 Nov 4;148:e270. doi: 10.1017/S0950268820002678 (PMC7674806; doi:10.1017/S0950268820002678)
Supplement: Supplementary file 1 [file S0950268820002678sup001.docx]

**Supplementary Material to**

**Dynamic Quarantine: A comparative analysis of the Chilean Public Health response to COVID-19**

Gonzalo Grebe^1^, Javier A. Velez^1^, Anton Tiutiunnyk^1^^[[1]](#footnote-1)^*, Diego Aragón-Caqueo^2^, JavierFernández-Salinas^2^, Mónica Navarrete^3^, David Laroze^1^

1 Instituto de Alta Investigación, CEDENNA, Universidad de Tarapacá, Casilla 7D, Arica,

Chile.

2 Escuela de Medicina, Universidad de Valparaíso, Valparaíso, Chile.

3 Escuela Universitaria de Administración y Negocios, EXPLORA, Universidad de Tarapacá,

Casilla 7D, Arica, Chile.

In this supplementary material we provide the information of different strategies of three country regarding to COVID-19based on the daily transmission rate (DTR). In addition, we provide the data of daily test in Chile until 15 May.

**I.- International response**

In this section, the responses of South Korea, Germany, as well as New Zealand, are analyzed. These three countries, with very different approaches, have achieved the relative control stage with a DTR of less than 2%, as shown in **Figure S1**.


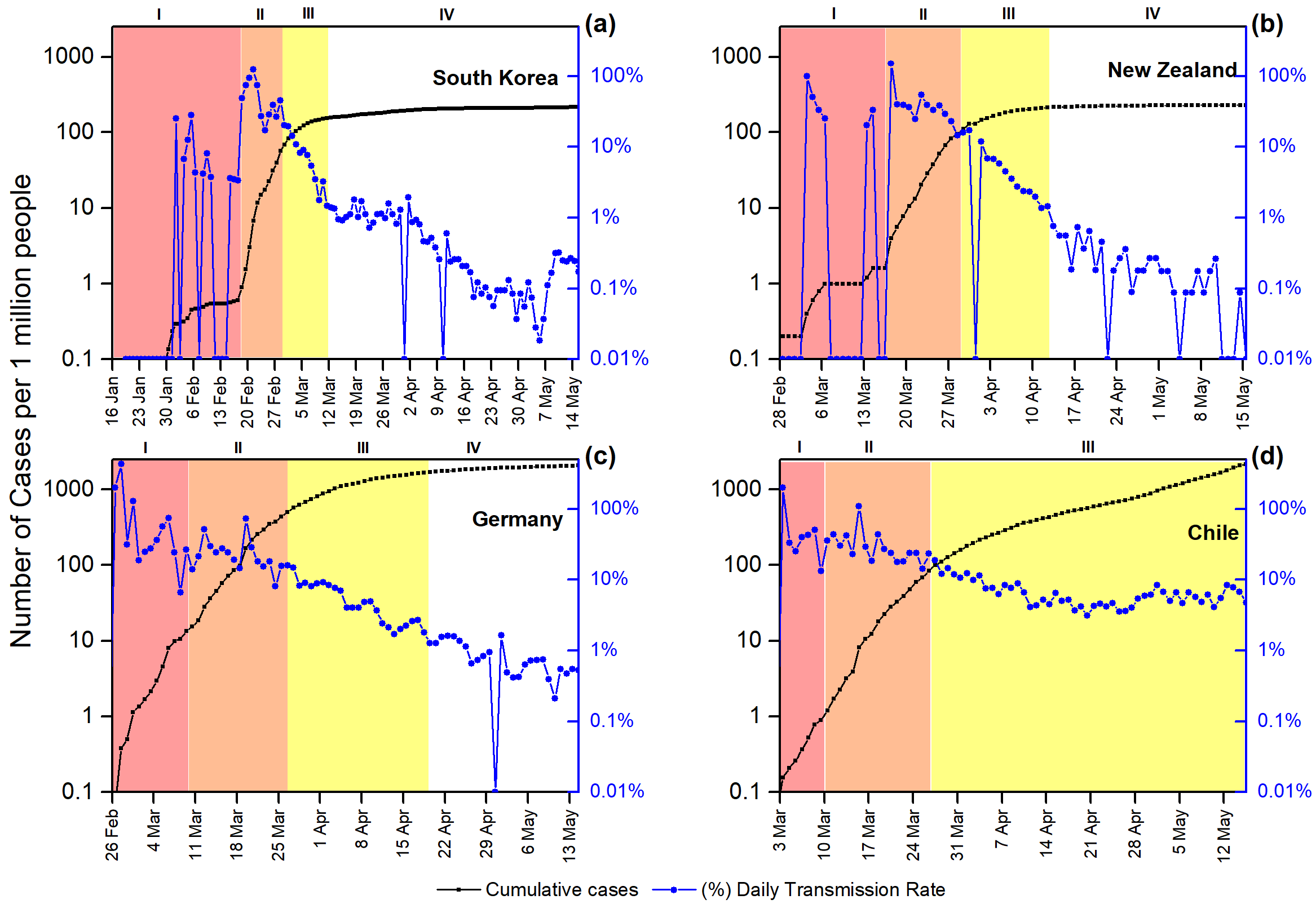


**Fig. S1:** Temporal evolution of the total number of cases (in black squares, logarithmic scale) and the DTR (in blue circles) from the first reported case in each country until 15 May. The different stages of the pandemic progression are color-coded as described above. **(a)** South Korea, first reported case on 20 January. (**b**) New Zealand, first reported case on 28 February. (**c**) Germany, first reported case on 26 February. (**d**) Chile, first reported case on 3 March. We remark that S1 is the same figure 1 of the manuscript.

**1.1 *South Korea***

South Korea was the 4th country to be affected by the virus, confirming its first case on 20 January 2020 [SM1], even before the World Health Organization (WHO) declared the outbreak as a Public Health Emergency of International Concern (PHEIC) [SM2]. Therefore, there was little evidence available on how to face this new threat and what strategies should be considered, besides the strict quarantine policy established in China by that time [SM3].

In contrast to China, the South Korean strategy did not include mandatory quarantine, emphasizing a policy of rapid and massive testing applied in the country, with a total daily testing capacity of more 15000 laboratory tests per day, with more than 600 testing stations where sometimes it is not even necessary to get out of the car to perform the test. It can be done under 20 minutes, averaging 10 minutes per person [SM4]. Furthermore, as of 15 May, it performs around 14 tests per 1000 people [SM5], with 753211tests performed up to date [SM6]. It can be inferred that the effectiveness of this policy lies in its massiveness and rapidness of the implemented response. It is widely known by now that the problem of contagion occurs when an infected individual circulates freely, further spreading the virus, without knowing it until it presents with the onset of symptoms if it does present. Therefore, easy access to testing, and rapid assessment of the consulting individual's state, a significant part of the problem is solved. The rapid tests allow to obtain a within minutes [SM7], giving a small window for further spreading in case individual isolation measures were not already taken.

On the other hand, although the procedure of PCR-based methods takes an estimate of 45 minutes to come up with a result [SM8], processing of the clinical samples and laboratory availability might delay the result to several days. This implies a larger window to spread the virus. Therefore, testing strategies should be applied [SM9].

The panel (**a**) of **Figure S1** shows the evolution over time of the number of cases per day (in black), and the DTR expressed as a percentage (in blue), respectively. The graph ranges from 20 January (since the first case was reported) until 15 May. The cases per day are expressed on a logarithmic scale on the left side, while the DTR scale expressed as a percentage is on the right side. It should be noted that the figure highlights in color code the different stages in the temporal evolution of the progression of the pandemic, using a degrade scale. In red, the stochastic uptake stage, in light red, the exponential growth stage, in yellow the intervention stage. Finally, the new growth regime is represented in white, which is a phase with a relative control.

It can be observed that since the first reported case on 20 January up until 18 February, 31 cases were reported; thus, the stochastic uptake stage can be established in that period. As mentioned earlier, South Korea was one of the first countries to be affected by the virus after China, thus, having a small window of time to prepare for this novel threat, besides that, the response was becoming a world-leading example. Since 19 February, with 46 cases, up until 29 February, a series of high DTR began, going from 46 cases to 2931in ten days, thus establishing the exponential growth phase. Since 1 March, the response's effect can be observed, lowering the DTR from 20% to 1.5% in 12 days. On 20 March, South Korea had already tested more than 370.000 individuals, and from there up until 15 May, the DTR stays between 1% to 0.1%. It is worth mentioning that 260 deaths were reported with a mortality rate of 2.36%, considerably below the 6.86% global average [SM10].

***1.2 New Zealand***

Another successful response was in New Zealand. This country was the second country besides China, and the first Western country to have relative control of the virus [SM11]. However, unlike South Korea, the first case was reported on 28 February [SM10]. Therefore, it had more information available in order to prepare the response. As compared to South Korea, its control policy was diametrically different, implementing a strict total quarantine at a country level [SM12]. This translates into the sustained result of the response strategy and the rapid establishment of relative control, as shown in the panel (**b**) of **Figure S1**. It can be inferred that the stochastic uptake stage begins with the first case reported on 28 February up until 17 March with 8 cases. Then, the exponential growth phase begins from 18 March to 28 March, where 408 new cases were reported in this 11-day window. The effect of the strategies applied can be observed as the DTR starts to decrease from 28 March up until 11 April progressively. Finally, the sustained stabilization began to reach 15 days, on 12 April, a 2% DTR. A new growth regime is achieved, with DTRs considerably below the 2% mark and some bordering the 0.1%. The strategy in New Zealand was a total countrywide quarantine established on 26 March when the curve was at the 262 confirmed cases. This quarantine was subsidized by the state, paying workers’ wages for up to 12 weeks [SM13]. Regarding the mortality rate, 21 deaths have been reported so far, being the last reported death on 6 May. This brings the mortality rate to 1.8%, considerably lower than 6.86% global average [SM10].

**1.3 *Germany***

The German response was based on a mixed strategy. It combined classic preventive and restrictive measures such as closing educational institutions and mass events, prohibiting meetings of more than two people but without going as far as total quarantine [SM12]. At the same time, extensive testing was implemented, performing as of 10 May of 2020, 3.15 million tests covering an estimate of 38 individuals per 1000 people [SM14, SM15]. As shown in panel (c) of **Figure S1**, the stochastic uptake stage started in late February to early March. From that point on, the exponential growth phase begins, rapidly increasing from a little over a thousand cases on 10 March to 42288 in 17 days (27 March) [SM10]. The effects of the strategies taken begin to be observed from 28 March, where the DTR starts to fall over time consistently. From there, it reaches the 2% mark on 14 April. From there on, the DTR roughly stays around this 2% mark. Finally, on 19 April, after 22 days, the new growth regime phase is achieved, where the DTR falls below the 2% mark and stays consistently below it, reaching and maintaining a DTR of 0.4% to date. Regarding the mortality of the outbreak, 7824 deaths were reported, which stands for 4.51% mortality rate, which is considerably lower than 6.86% global average [SM10].

**II. Daily COVID-19 test per thousand people in Chile**

**Figure S2** shows the daily tests per thousand people with 7-day smoothed representation. We remark that the testing in Chile raised from 0.17 to 0.64 for every 1000 people from 8 April to 15 May [SM16]. In this time interval Chile has the higher testing rate in Latin America [SM17].

**Fig. S2**: Temporal evolution of the daily tests per thousand people in Chile.

**References**

1. World Health Organization (WHO). Novel Coronavirus (2019- nCoV). Situation report – 1. Geneva: WHO; January 21, 2020. Available from: <https://www.who.int/docs/default-source/coronaviruse/situation-reports/20200121-sitrep-1-2019-ncov.pdf?sfvrsn=20a99c10_4>
2. World Health Organization (WHO). Statement on the Second Meeting of the International Health Regulations (2005) Emergency Committee Regarding the Outbreak of Novel Coronavirus (2019-NCoV). Geneva: WHO; January 30, 2020. Available from: <https://www.who.int/news-room/detail/30-01-2020-statement-on-the-second-meeting-of-the-international-health-regulations-(2005)-emergency-committee-regarding-the-outbreak-of-novel-coronavirus-(2019-ncov)>
3. World Health Organization (WHO). Report of the WHO-China Joint Mission on Coronavirus Disease 2019 (COVID-19). Geneva: WHO; February 24, 2020. Available from:
   <https://www.who.int/docs/default-source/coronaviruse/who-china-joint-mission-on-covid-19---final-report-1100hr-28feb2020-11mar-update.pdf?sfvrsn=1a13fda0_2>
4. Lee D, Lee J. Testing on the Move South Korea’s rapid response to the COVID-19 pandemic. *Transportation Research Interdisciplinary Perspectives*. Apr 21, 2020. Available from: doi: 10.1016/j.trip.2020.100111.
5. Our World in Data. Total COVID-19 tests per 1000 people, South Korea. Oxford Martin School. Available from: <https://ourworldindata.org/coronavirus-testing#south-korea> [Accessed May 15, 2020]
6. Ministry of Health and Welfare. Cases in Korea. Coronavirus Disease-19 (COVID-19), Republic of Korea. Available from: <http://ncov.mohw.go.kr/en/> [Accessed May 15, 2020]
7. Li Z, *et al*. Development and clinical application of a rapid IgM‐IgG combined antibody test for SARS‐CoV‐2 infection diagnosis. *J Med Virol* 2020. Available from: <https://doi.org/10.1002/jmv.25727>
8. Udugama B, *et al*. Diagnosing COVID-19: The Disease and Tools for Detection. *ACS nano* 2020. Available from: https://doi.org/10.1021/acsnano.0c02624
9. Aragón‐Caqueo D, Fernández‐Salinas J, Laroze D. Optimization of group size in pool testing strategy for SARS‐CoV‐2: A simple mathematical model. *J Med Virol* 2020. Available from: <https://doi.org/10.1002/jmv.25929>
10. European Centre for Disease Prevention and Control. Today’s data on the geographic distribution of COVID-19 cases worldwide as of 15 May 2020. Available from: <https://www.ecdc.europa.eu/en/publications-data/download-todays-data-geographic-distribution-covid-19-cases-worldwide> [Accessed May 15, 2020]
11. Our World in Data. Daily vs. Total confirmed COVID-19 cases, New Zealand. Oxford Martin School. Available from: <https://ourworldindata.org/grapher/covid-daily-vs-total-cases?country=NZL> [Accessed May 15, 2020]
12. International Monetary Fund. Policy Responses to COVID19. Policy Tracker. Available from: <https://www.imf.org/en/Topics/imf-and-covid19/Policy-Responses-to-COVID-19> [Accessed May 15th 2020].
13. Ministry of Business, Innovation & Employment of New Zealand. Wage Subsidy Scheme. Wellington: Employment New Zealand. 14 May 2020. Available from: <https://www.employment.govt.nz/leave-and-holidays/other-types-of-leave/coronavirus-workplace/wage-subsidy/>
14. Statista. Coronavirus (COVID-19) tests performed in Europe as of May 13, 2020, by country. 2020. Available from: <https://www.statista.com/statistics/1109066/coronavirus-testing-in-europe-by-country/> [Accessed May 15 2020]
15. Our World in Data. Total COVID-19 tests per 1000 people, Germany. Oxford Martin School. Available from: <https://ourworldindata.org/grapher/full-list-cumulative-total-tests-per-thousand?time=..&country=DEU> [Accessed May 15, 2020]
16. OurWorld in Data Total COVID-19. Available: <https://ourworldindata.org/grapher/daily-tests-per-thousand-people-smoothed-7-day?time=2020-04-15..2020-05-15&country=ARG~BRA~COL~ECU~MEX~PAN~PER~BOL~CHL~CRI~CUB~SLV~PRY~URY>[Accessed May 15, 2020]

1. *[tyutyunnyk.a.m@academicos.uta.cl](mailto:tyutyunnyk.a.m@academicos.uta.cl) [↑](#footnote-ref-1)
